# sFlt-1 impairs neurite growth and neuronal differentiation in SH-SY5Y cells and human neurons

**DOI:** 10.1042/BSR20240562

**Published:** 2024-05-17

**Authors:** Aaron Barron, Lauren Barrett, Jetro J. Tuulari, Linnea Karlsson, Hasse Karlsson, Cathal M. McCarthy, Gerard W. O'Keeffe

**Affiliations:** 1 Department of Anatomy and Neuroscience, University College, Cork, Ireland; 2 Department of Pharmacology and Therapeutics, University College Cork, Cork, Ireland; 3 FinnBrain Birth Cohort Study, Turku Brain and Mind Centre, Department of Clinical Medicine, University of Turku, Turku, Finland; 4 Department of Psychiatry and Turku Brain and Mind Centre, University of Turku and Turku University Hospital, Turku, Finland; 5 Turku Collegium for Science, Medicine and Technology, University of Turku, Turku, Finland; 6 Centre for Population Health Research, University of Turku, Turku University Hospital, Turku, Finland; 7 Department of Clinical Medicine, Paediatrics and Adolescent Medicine, Turku University Hospital and University of Turku, Turku, Finland; 8 Department of Clinical Medicine, Unit of Public Health, University of Turku, Turku, Finland

**Keywords:** axon, differentiation, growth, Neuron, Pre-eclampsia, sFLT-1

## Abstract

Pre-eclampsia (PE) is a hypertensive disorder of pregnancy which is associated with increased risk of neurodevelopmental disorders in exposed offspring. The pathophysiological mechanisms mediating this relationship are currently unknown, and one potential candidate is the anti-angiogenic factor soluble Fms-like tyrosine kinase 1 (sFlt-1), which is highly elevated in PE. While sFlt-1 can impair angiogenesis via inhibition of VEGFA signalling, it is unclear whether it can directly affect neuronal development independently of its effects on the vasculature. To test this hypothesis, the current study differentiated the human neural progenitor cell (NPC) line ReNcell® VM into a mixed culture of mature neurons and glia, and exposed them to sFlt-1 during development. Outcomes measured were neurite growth, cytotoxicity, mRNA expression of *nestin*, *MBP*, *GFAP*, and *βIII-tubulin*, and neurosphere differentiation. sFlt-1 induced a significant reduction in neurite growth and this effect was timing- and dose-dependent up to 100 ng/ml, with no effect on cytotoxicity. sFlt-1 (100 ng/ml) also reduced *βIII-tubulin* mRNA and neuronal differentiation of neurospheres. Undifferentiated NPCs and mature neurons/glia expressed *VEGFA* and *VEGFR-2*, required for endogenous autocrine and paracrine VEGFA signalling, while sFlt-1 treatment prevented the neurogenic effects of exogenous VEGFA. Overall, these data provide the first experimental evidence for a direct effect of sFlt-1 on neurite growth and neuronal differentiation in human neurons through inhibition of VEGFA signalling, clarifying our understanding of the potential role of sFlt-1 as a mechanism by which PE can affect neuronal development.

## Introduction

Pre-eclampsia (PE) is a commonly occurring hypertensive disorder of pregnancy (HDP) which affects approximately 5% of first-time pregnancies, and is characterised by new-onset hypertension on or after 20 weeks’ gestation and one or more of proteinuria, organ dysfunction or uteroplacental dysfunction [1,2]. While PE has long been known to predispose the mother towards long-term morbidity, it is now recognised to have deleterious consequences for the long-term health outcomes of the offspring [3,4]. Of particular interest, PE exposure increases the risk of diagnosis of neurodevelopmental disorders, particularly autism spectrum disorder (ASD), attention-deficit/hyperactivity disorder (ADHD), and intellectual disability (ID) [5–8]. In line with this, children who are prenatally exposed to PE exhibit neuroanatomical alterations [9,10], and offspring from animal models of PE exhibit various brain and behavioural deficits [11–13]. PE exposure is, therefore, likely to influence neurodevelopmental processes in the foetal brain such as differentiation of neural progenitor cells (NPCs) into neurons, or neurite growth, both of which have been implicated in neurodevelopmental disorders [14,15]. Currently, however, the cellular and molecular mechanisms by which PE affects neuronal development are yet to be elucidated.

PE involves various pathophysiological pathways that could each be responsible for the effects on foetal neurodevelopment – these include maternal immune activation [16], oxidative stress [17,18], and altered placental secretion of neurotrophic factors [19]. Overall, however, there is a lack of direct experimental evidence for the molecular mediators involved. Arguably the most well described molecular alteration in PE is an increase in the anti-angiogenic factor soluble Fms-like tyrosine kinase 1 (sFlt-1), also known as soluble vascular endothelial growth factor receptor 1 (sVEGFR-1) [20–22]. Elevated sFlt-1 circulates in PE and contributes to endothelial cell dysfunction and disease progression, and is so well characterised that the elevated sFlt-1 and corresponding decrease in pro-angiogenic placental growth factor (PlGF) is being used clinically as a potential diagnostic and prognostic biomarker of PE [23,24]. Furthermore, overexpression of human sFlt-1 is used as a pre-clinical animal model of PE, and knockdown of *sFlt-1* reduces the features of PE in a baboon PE model [25,26].

Importantly, sFlt-1 is elevated in the umbilical cord blood of PE-exposed infants, and therefore may be increased in the developing foetal brain [21,27,28]. It has been hypothesised that the imbalance in angiogenic factors in PE, including elevated sFlt-1 and reduced PlGF, could impair the normal development of the cerebrovasculature [29], and there is in fact evidence of abnormal cerebral blood vessel development in children prenatally exposed to PE [10]. It is less well known, however, whether elevated sFlt-1 can directly affects neuronal development. sFlt-1 circulates freely and binds the angiogenic factor VEGFA with high affinity, sequestering it to prevent pro-angiogenic signalling through the transmembrane tyrosine kinase receptor, VEGFR-2 [30,31]. The trophic effects of VEGFA are not confined to the vasculature; however, it also exerts a neurotrophic effect on developing neurons. Animal studies have demonstrated a neurogenic role for VEGFA *in vivo* [32–34]. Although the study by Okabe [2020] observed the effects of conditional VEGFA knockout on the brain were through indirect effects on cerebral blood vessel development, several studies have shown a potent and direct neurogenic effect of VEGFA in neuronal culture systems devoid of endothelial cells, whereby it promotes neural progenitor cell proliferation, neurite growth, MAP-2 and βIII Tubulin expression, and activation of neurogenic signalling pathways [32,33,35–37]. Neurons do not rely exclusively on exogenous VEGFA, but synthesise their own *de novo*, and express the VEGFR-2 receptor to mediate downstream signalling [32,33,36]. This is highly suggestive that, independently of the vasculature, neurons can engage in neurogenic autocrine and paracrine VEGFA signalling, which may therefore be affected by elevated levels of s-Flt1.

In support of this, a recent transgenic mouse study reported that fetoplacental sFlt-1 overexpression alters neuronal cell density in the cortex and caudate putamen of the offspring, and reduces cerebral *Map2* and *Ngf* expression [25]. Another pair of studies conditionally overexpressed sFlt-1 in the brain of adult mice, and examined the two adult neural stem cell niches, the subventricular zone and subgranular zone. Local sFlt-1 overexpression impaired the development and maturation of adult newly-born olfactory interneurons, but not hippocampal neurons [38,39]. This raises the possibility that different subpopulations of neurons differ in their responsiveness to sFlt-1, and it is unclear how much of the *in vivo* effect is indirect, via paracrine signalling through other cells such as ependymal or microglial cells. To date, only two studies have examined a direct effect of sFlt-1 in neurons, whereby sFlt-1 reduced NPC proliferation and increased apoptosis [32,37], although in the 20 years since their publication, it has not been shown whether sFlt-1 directly affects neurogenesis or neuronal morphology. Furthermore, the entire literature on VEGFA and sFlt-1 *in vitro* and *in vivo* is confined to rodent neurons, and thus it is currently unclear to what extent a potential anti-neurogenic effect of sFlt-1 might be conserved in humans, owing to species differences in gene expression profiles, electrophysiology, and developmental rates [40,41]. The current study sought to test the hypothesis that exogenous sFlt-1 exerts anti-neurogenic, and neurite growth inhibitory effects in developing human neurons; and, secondly, that these effects are mediated through inhibition of endogenous VEGFA signalling.

## Materials and methods

### Monolayer cell culture and treatments

Human neural progenitor ReNcell® VM cells (Sigma Aldrich) were cultured in Dulbecco’s modified Eagle’s (DMEM)/Nutrient Mixture F-12 Ham’s medium without antibiotics, supplemented with 2 mM L-glutamine, 10 U/ml heparin, 2% (v/v) B-27, 1% (v/v) N-2, and 20 ng/ml EGF and 20 ng/ml FGF-2, and maintained in a T75 culture flask (Sarstedt) at 37°C and 5% CO_2_. B-27 and N-2 from ThermoFisher, FGF-2 from Peprotech, all other reagents from Sigma Aldrich. Media was changed every 2 days and when 80% confluent, cells were passaged and seeded at 12,500 cells per well in a laminin-coated 48-well plate for immunocytochemistry, or at 1 × 10^6^ cells per well in a laminin-coated 6-well plate for RNA extraction. Twenty-four hours after plating, cells were washed with HBSS, and fresh media was added without EGF and FGF-2 to initiate differentiation [42]. Cells were differentiated for 15 days *in vitro* (DIV), and a complete media change was performed every 3 days.

For all experiments, treatments were given after each media change beginning on day 1 of differentiation, and analyses were performed 72 h after the last treatment. Final concentrations used were 0.1, 1, 10, and 100 ng/ml sFlt-1 (Abcam), and 50 ng/ml VEGFA (Peprotech). Sterile PBS was used in all experiments as a vehicle control.

Human neuroblastoma SH-SY5Y cells (ATCC) were cultured in Dulbecco’s modified Eagle’s (DMEM)/Nutrient Mixture F-12 Ham’s medium, supplemented with 2 mM L-glutamine, 1% penicillin-streptomycin, and 10% FBS (all from Sigma Aldrich), and maintained in a T75 culture flask (Sarstedt) at 37°C and 5% CO_2_. Media was changed every 3 days and when 80% confluent, cells were passaged and seeded at 25,000 cells per well in a 24-well plate. Twenty-four hours after plating, treatments began. Cells were treated daily with 10 μM retinoic acid (RA, Sigma Aldrich) for 3 days to induce partial neuronal differentiation, concomitant with sFlt-1 treatment, and analyses were performed 72 h after first treatment.

### Neurosphere cell culture

For neurosphere culture, ReNcell® VM underwent 7 days of proliferation followed by 4 days of differentiation. During the proliferation phase, 10^6^ cells were cultured in suspension in Dulbecco’s modified Eagle’s (DMEM)/Nutrient Mixture F-12 Ham’s medium without antibiotics, supplemented with 2 mM L-glutamine, 10 U/ml heparin, 2% B-27, 1% N-2, and 20 ng/mL EGF and FGF2 in an uncoated T25 flask (Sarstedt) and maintained at 37°C and 5% CO_2_ with shaking. Cells were treated daily with 20 ng/ml EGF and FGF2 to prevent spontaneous differentiation, and on day 4 given a partial media change and treatment. Neurospheres were imaged by phase contrast microscopy at 4 DIV and 7 DIV to measure neurosphere diameter during proliferation stage.

Equal numbers of neurospheres were seeded onto laminin-coated 12-well plates in media without EGF or FGF2 to initiate differentiation. During this stage, cells migrate rapidly from the core of the sphere, grow extensive neurites, and differentiate into neurons and glia. Two alternative experiments were conducted, with cells exposed to 100 ng/ml sFlt-1 either for the full 11 days (treatment on days 1 and 4 of proliferation and day 1 of differentiation), or for the 4-day differentiation stage only (treatment on day 1 of differentiation). In both cases, analyses were performed at DIV 11 (7 days proliferation and 4 days differentiation).

### Cytotoxicity assay

Cytotoxicity was assessed using the CyQUANT^™^ LDH Cytotoxicity Assay Kit (Invitrogen). The LDH assay is a colorimetric method which measures extracellular lactate dehydrogenase (LDH) activity to determine cytotoxic cell damage. As per manufacturer’s guidelines, culture media from each experiment was collected and centrifuged to remove any remaining cells or debris, and the supernatant was retained and used for the assay. Approximately 50 μl of the medium was combined with 50 μl of the reaction mixture in a flat-bottomed, 96-well plate and incubated for 30 min at room temperature in darkness. Approximately 50 μl of stop solution was used to terminate the reaction, and absorbance was measured at 680 nm and subtracted from absorbance at 490 nm.

### Immunocytochemistry

Monolayer and neurosphere cultures were fixed in 4% paraformaldehyde (PFA) at experimental endpoint and preserved in 10 mM PBS with 0.02% -Triton-X (PBS-T). Cells were incubated in 5% BSA at room temperature for 1 h to block non- specific binding and incubated at 4°C overnight with a primary antibody against βIII tubulin (1:1000, R&D Systems MAB1195). After overnight incubation, cells were washed in PBS-T and incubated at room temperature for 2 h with goat anti-mouse Alexa Fluor 488 secondary antibody (1:500, Invitrogen A11001). Cells were then re-blocked and stained overnight with a primary antibody against GFAP (1:1000, Abcam ab68428), followed by 2 h goat anti-rabbit Alexa Fluor 594 secondary antibody (1:500, Invitrogen A11012). Cells were washed in PBS-T, counterstained with DAPI, and imaged by fluorescence microscopy.

### Microscopy and image analysis

All cells were imaged at ×20 magnification on an Olympus IX71 inverted microscope with a DP72 camera. SH-SY5Y cells and neurospheres at DIV 4 and DIV 7 were imaged live under phase contrast light microscopy; immunostained ReNcell® VM cells were imaged by fluorescence microscopy with FITC, DAPI and TXRED filters. Five non-overlapping fields were acquired per well and analyzed using ImageJ. Neurites were traced to calculate neurite length; in the case of ReNcell® VM cells, neurites were measured only in cells which were βIII tubulin+ and GFAP-. For positivity analysis, composite images were created, and a sample field of view was counted for cells which were βIII tubulin+/GFAP-, βIII tubulin-/GFAP+, βIII tubulin+/GFAP+, or βIII tubulin-/GFAP-. All experiments were performed in a blinded fashion.

### RNA extraction, reverse transcription and polymerase chain reaction

RNA was extracted from confluent monolayers of ReNcell® VM using QIAzol lysis reagent and RNeasy Plus Universal RNA Isolation kit (Qiagen) as per manufacturer’s guidelines. RNA was then quantified by NanoDrop spectrophotometer (ThermoFisher) and stored at −80°C until further analysis. Āpproximately 500 ng RNA per sample was used to synthesise complementary DNA (cDNA) using High-Capacity cDNA Reverse Transcription Kit (ThermoFisher) as per manufacturer’s guidelines. cDNA was stored at 4°C until further use.

mRNA expression was determined by quantitative polymerase chain reaction (qPCR). Single-gene reaction mixtures were made as per manufacturer’s instructions using cDNA samples, RNase-free water, 2X TaqMan® Gene Expression Master Mix (ThermoFisher), and 20X TaqMan® Gene Expression Assays (ThermoFisher). The following FAM™-labelled probes were used: β2 Microglobulin (B2M) (Hs00187842_m1), Nestin (Hs04187831_g1), βIII tubulin (Hs00801390_s1), GFAP (Hs00909233_m1), Meylin Basic Protein (MBP) (Hs00921945_m1), VEGFA (Hs00900055_m1), VEGFR-1/sFlt-1 (Hs01052961_m1), and VEGFR-2 (Hs00911700_m1). cDNA was amplified for 40 cycles using the Light Cycler 480 thermocycler (Roche). The cycle threshold (*C*t) for detection of fluorescent signal of each gene was normalised to that of B2M as an endogenous control and used to calculate the delta-delta (dd) *C*t, expressed as the relative change in expression compared with the control group mean.

### Statistical analysis

All statistical analyses were performed using Graphpad Prism 9. Statistical significance (α) was set at *P*<0.05, and the statistical tests applied to the data were one-way ANOVA followed by Dunnett’s post-hoc test, Student’s paired two-tailed *t*-test, and two-way ANOVA, followed by Fisher’s least significant difference (LSD) post-hoc test. All data are expressed as the mean with standard error of the mean (SEM) where indicated.

## Results

### sFlt-1 reduces neurite growth in RA-differentiated SH-SY5Y cells and human NPC-derived neurons

**Figure 1:**
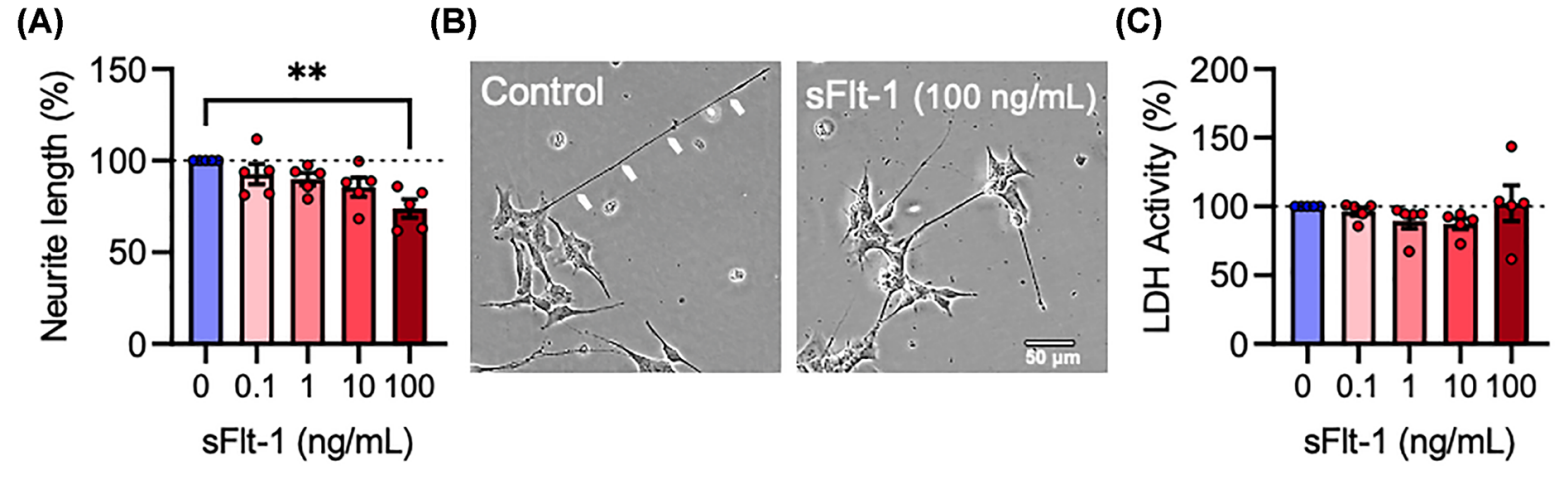
sFlt-1 reduces neurite growth in RA-differentiated SH-SY5Y cells RA-differentiated SH-SY5Y cells were treated with 0.1, 1, 10, or 100 ng/ml sFlt-1 or PBS as vehicle control, daily for 72 h. (**A**) Graph and (**B**) representative photomicrographs of neurite length. (**C**) Graph of extracellular LDH activity as a measure of cytotoxicity. Data are mean + SEM from five independent experiments (*n*=5) expressed as percentage of the control. One-way ANOVA and post-hoc Dunnett’s test (**P<0.01 vs. control).

SH-SY5Y neuroblastoma cells were differentiated with RA and exposed to 0, 0.1, 1, 10, and 100 ng/ml sFlt-1 daily for 3 days to determine whether sFlt-1 affected neurite growth or cytotoxicity. sFlt-1 elicited a dose-dependent reduction in neurite growth (F_4,20_ = 5.042, *P*<0.01), which was statistically significant only at the highest concentration of 100 ng/ml (*P*<0.01) with a 25% reduction in neurite length (Figure 1A,B). sFlt-1 had no cytotoxic effect at any concentration tested (F_4,20_ = 0.9528, *P*=0.455) (Figure 1C).

**Figure 2:**
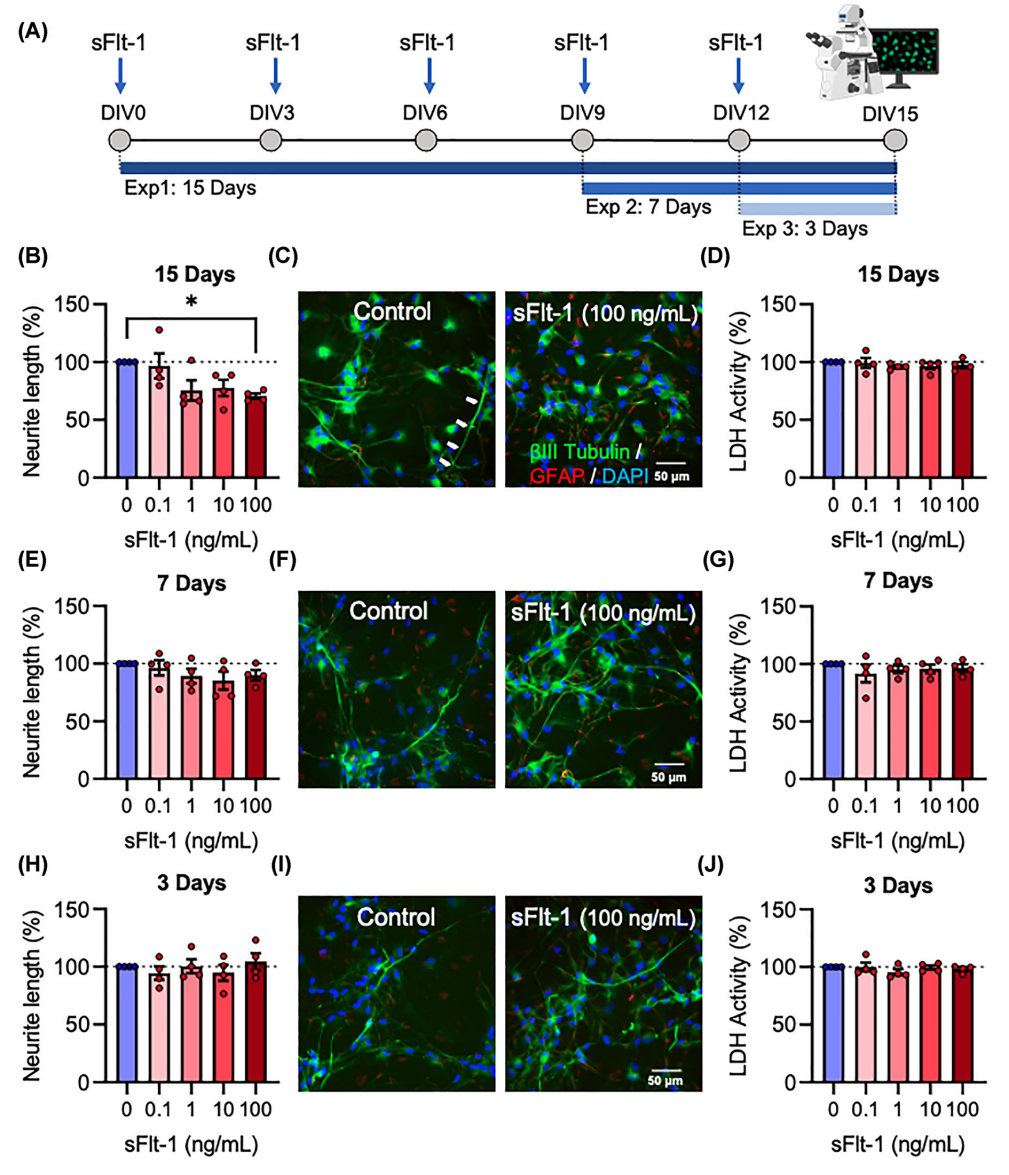
sFlt-1 reduces neurite growth in human NPC-derived neurons ReNcell® VM were differentiated from human neural progenitor cells into neurons and glia over 15 days while exposed to 0.1, 1, 10, or 100 ng/ml sFlt-1, or PBS as vehicle control. (**A**) Timeline of cell treatments: all cells received a media change every 72 h for 15 days, followed by treatment with relevant concentration of sFlt-1. Three alternative experiments were conducted, with cells exposed to sFlt-1 for the full 15 days’ differentiation, the last 7 days, or the last 3 days. (**B,C**) Graph and representative photomicrographs of neurite length and (**D**) cytotoxicity in cells treated with sFlt-1 for 15 days. (**E,F**) Graph and representative photomicrographs of neurite length and (**G**) cytotoxicity in cells treated with sFlt-1 for 7 days. (**H,I**) Graph and representative photomicrographs of neurite length and (**J**) cytotoxicity in cells treated with sFlt-1 for 3 days. Data are mean + SEM from four independent experiments (*n*=4) expressed as percentage of the control. One-way ANOVA and post-hoc Dunnett’s test (*P<0.05 vs. control); DIV = days* in vitro*.

To determine whether this phenotype was present in mature human neurons, the dose response experiment was repeated in ReNcell® VM neural progenitor cells as they differentiated over 15 days into a mixed culture of neurons and glia. Three alternative experiments were conducted, with cells exposed to sFlt-1 for the full 15 days’ differentiation, the last 7 days, or the last 3 days only (Figure 2A). When exposed for the full 15 days’ differentiation, sFlt-1 reduced neurite growth (F_4,15_ = 3.57, *P*<0.05), which was only statistically significant at 100 ng/ml (*P*<0.05) (Figure 2B,C) with a 30% reduction in neurite length, recapitulating the inhibition of neurite growth observed in SH-SY5Y cells. When exposed only for the last 7 days, however, the reduction was not statistically significant (F_4,15_ = 1.082, *P*=0.4) (Figure 2E,F), and when exposed only for the last 3 days, there was no observable effect at all (F_4,15_ = 0.5025, *P*=0.735) (Figure 2H,I). sFlt-1 was not cytotoxic at any concentration after 15 (F_4,15_ = 0.4607, *P*=0.764), 7 (F_4,15_ = 0.4624, *P*=0.762), or 3 (F_4,15_ = 0.7331, *P*=0.583) days’ exposure (Figure 2D,G,J). Taken together, these data provide evidence that sFlt-1 reduces neurite growth in developing human neurons, but only at long-term exposure, without any accompanying toxic effects on the cells.

### sFlt-1 reduces neuronal differentiation of human NPCs

**Figure 3:**
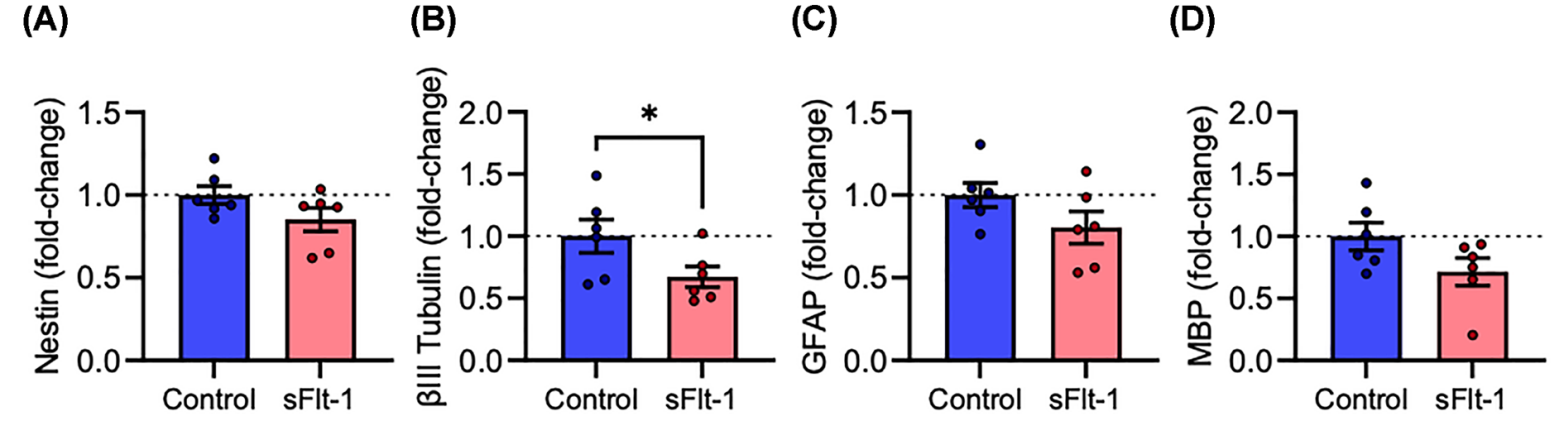
sFlt-1 reduces βIII tubulin mRNA expression in human NPC-derived neurons ReNcell® VM were differentiated into neurons and glia over 15 days while exposed to 100 ng/ml sFlt-1 or PBS as vehicle control. On day 15, RNA was extracted, and relative mRNA expression measured by RT-qPCR. Graphs of (**A**) Nestin, (**B**) βIII Tubulin, (**C**) GFAP, and (**D**) MBP mRNA expression. Data are mean + SEM from six independent experiments (*n*=6) expressed as fold-change of the control group mean. Student’s paired *t*-test (*P<0.05 vs. control).

ReNcell® VM neural progenitor cells were treated with 100 ng/ml sFlt-1 during 15 days’ differentiation into neurons and glia, and RNA was extracted to assess gene expression by RT-qPCR. sFlt-1 did not affect expression of *Nestin* mRNA (*t*_5_ = 1.423, *P*=0.214), a common marker for neural progenitor cells (Figure 3A). sFlt-1-treated cells expressed lower levels of *βIII-tubulin* mRNA (*t*_5_ = 2.907, *P*<0.05), a neuronal cytoskeletal protein (Figure 3B), but not the glial markers *GFAP* (*t*_5_ = 1.343, *P*=0.237) (Figure 3C) or *MBP* (*t*_5_ = 1.698, *P*=0.1502) (Figure 3D).

**Figure 4:**
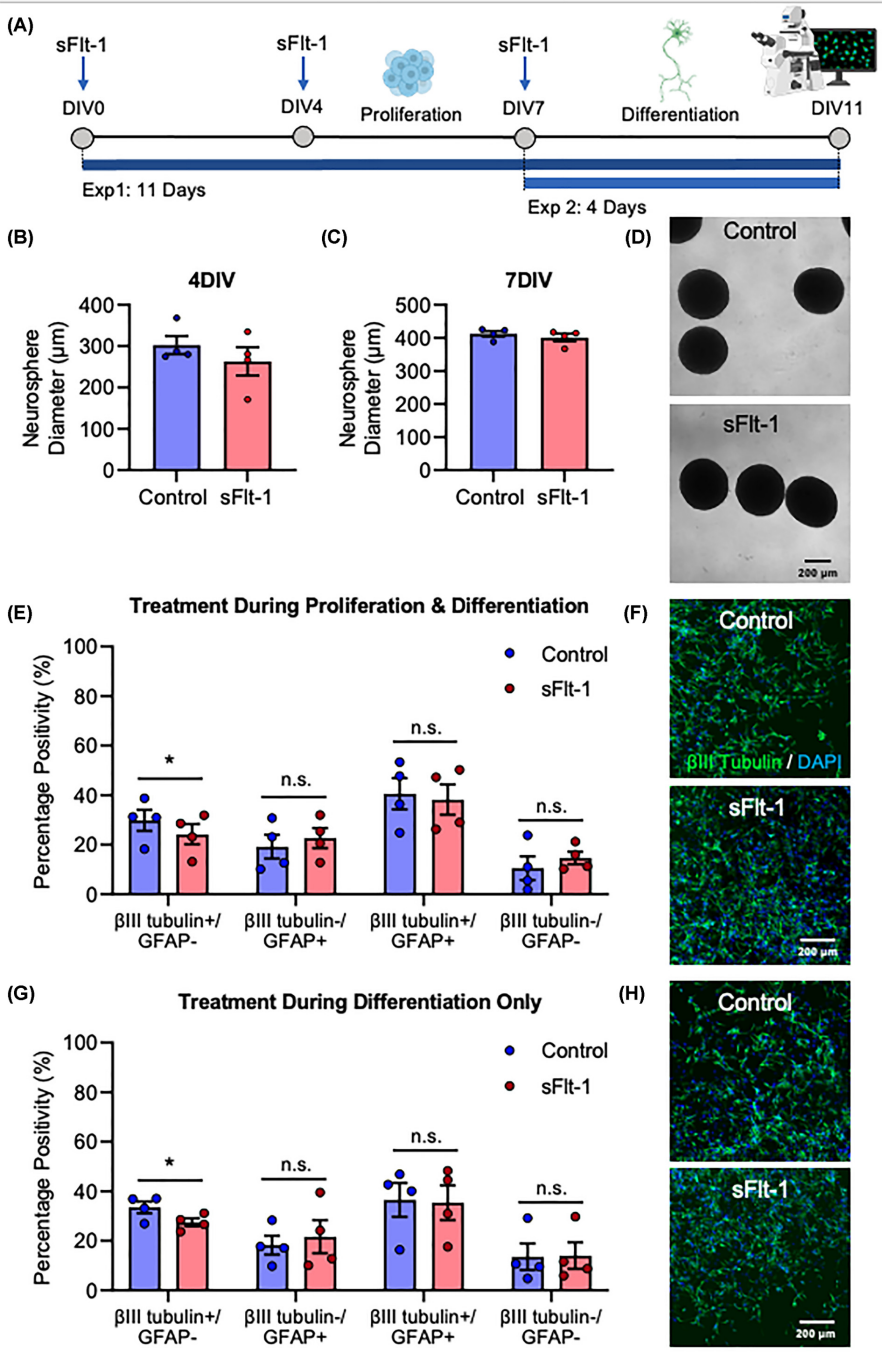
sFlt-1 reduces neuronal differentiation of human neurospheres ReNcell® VM were grown into neurospheres and exposed to 100 ng/ml sFlt-1 or PBS as vehicle control. (**A**) Timeline of cell treatments: neurospheres were cultured in suspension with neural progenitors kept in a proliferative state for 7 days, followed by differentiation for 4 days. Two alternative experiments were conducted, with cells exposed to sFlt-1 either for the full 11 days, or for the 4-day differentiation stage only. (**B,C**) Neurosphere diameter at 4DIV and 7DIV, and (**D**) representative photomicrographs at 7DIV. (**E,F**) Graph and representative photomicrographs of βIII Tubulin and GFAP protein expression in cells treated for both proliferation and differentiation stages. (**G,H**) Graph and representative photomicrographs of βIII Tubulin and GFAP protein expression in cells treated for differentiation stage only. Data are mean + SEM from four independent experiments (*n*=4) expressed as percentage of positive cells. Student’s paired *t*-test (*P<0.05 vs. control).

To determine whether this reduction in *βIII-tubulin* mRNA expression is reflected at the protein level, ReNcell® VM were grown as neurospheres, which involved a 7-day proliferation and a 4-day differentiation stage, and immunostained for βIII-tubulin and GFAP. Neurospheres were exposed to 100 ng/ml sFlt-1 either for both stages, or for the proliferation or differentiation stages only (Figure 4A). Exposure to sFlt-1 during proliferation did not affect neurosphere diameter at 4 DIV (*t*_3_ = 1.590, *P*=0.21) or 7DIV (*t*_3_ = 0.761, *P*=0.502) (Figure 4B–D). However, sFlt-1 did decrease neuronal differentiation. Exposure for the full 11 days reduced the proportion of cells which expressed βIII-tubulin but not GFAP (*t*_3_ = 4.527, *P*<0.05), representing mostly mature neurons which no longer express GFAP. There was a correspondingly small but statistically non-significant increase in cells expressing GFAP but not βIII-tubulin (*t*_3_ = 2.336, *P*=0.102), and no difference in the number of cells expressing both βIII-tubulin and GFAP (*t*_3_ = 0.9217, *P*=0.425), or those expressing neither protein (*t*_3_ = 1.136, *P*=0.338) (Figure 4E,F). Exposure to sFlt-1 for only the 4-day differentiation stage was sufficient to produce this phenotype, reducing the number of βIII-tubulin+/GFAP- cells (*t*_3_ = 5.577, *P*<0.05), but not the number of βIII-tubulin-/GFAP+ (t_3_ = 0.8843, *P*=0.443), βIII-tubulin+/GFAP+ (*t*_3_ = 0.4369, *P*=0.692), or βIII-tubulin-/GFAP- (*t*_3_ = 1.038, *P*=0.378) cells (Figure 4G,H). Lastly, sFlt-1 treatment reduced neurite length from the neurospheres if added during both stages (*t*_3_ = 4.003, *P*<0.05) or only the proliferation stage (*t*_3_ = 3.2984, *P*<0.05), but not if added only during the differentiation stage (*t*_3_ = 0.34519, *P*=0.753), which is somewhat in line with the previous finding in monolayer cultures whereby sFlt-1 reduced neurite growth only if it was present from the beginning of the experiment. Overall, these findings demonstrate that exposure of developing human neurons to sFlt-1 reduces their βIII-tubulin mRNA expression, and the proportion of cells which express βIII-tubulin protein but not GFAP, suggesting an anti-neurogenic role for sFlt-1.

### ReNcell VM express machinery for endogenous VEGFA autocrine and paracrine signalling, which is unchanged by sFlt-1 treatment

**Figure 5:**
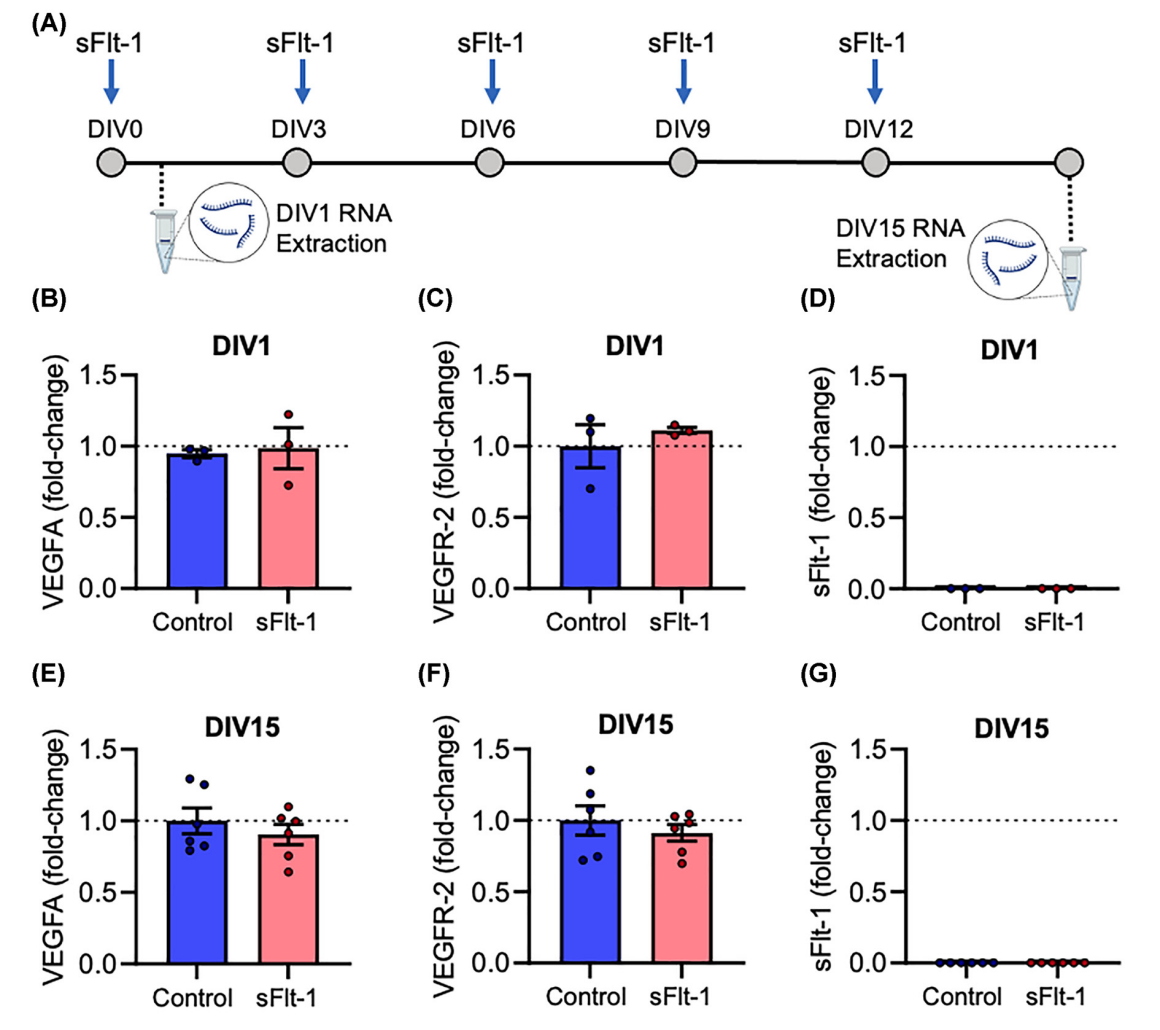
ReNcell VM express machinery for endogenous VEGFA autocrine and paracrine signalling, which is unchanged by sFlt-1 treatment ReNcell® VM were differentiated into neurons and glia over 15 days while exposed to 100 ng/ml sFlt-1, or PBS as vehicle control. (**A**) Timeline of experiment: on day 1 (undifferentiated) and day 15 (differentiated), RNA was extracted, and relative mRNA expression measured by RT-qPCR. (**B–D**) Graphs of (B) VEGFA, (C) VEGFR-2, and (D) sFlt-1/VEGFR-1 mRNA expression at DIV1. (**E–G**) Graphs of (E) VEGFA, (F) VEGFR-2, and (G) sFlt-1/VEGFR-1 mRNA expression at DIV15. Data are mean + SEM from three independent experiments (*n*=3) expressed as fold-change of the control group mean. Student’s paired *t*-test.

sFlt-1 is known to act as a decoy receptor to VEGFA; however, there is no VEGFA in the media used in the present study. We, therefore, tested the hypothesis that ReNcell® VM express endogenous VEGFA and the VEGFR-2 receptor required for autocrine and paracrine VEGFA signalling, as a potential mechanism by which sFlt-1 could exert its anti-neurogenic effects. RNA was extracted from both undifferentiated NPCs (DIV 1) and mature neurons and glia (DIV 15) (Figure 5A). At DIV1, NPCs express both *VEGFA* and *VEGFR-2* mRNA, and the relative expression of both genes was unaffected by sFlt-1 treatment (*VEGFA*, *t*_2_ = 0.2296, *P*=0.84; *VEGFR-2*, *t*_2_ = 0.7554, *P*=0.529) (Figure 5B,C). The expression of both genes was sustained at DIV15 after neuronal differentiation, and was unchanged by sFlt-1 (*VEGFA*, *t*_5_ = 0.6177, *P*=0.564; *VEGFR-*2, *t*_5_ = 0.5996, *P*=0.575) (Figure 5E,F). Therefore, both undifferentiated human NPCs, and differentiated human neurons and glia, express both the ligand and receptor for autocrine and paracrine VEGFA signalling. Importantly, no *VEGFR-1/sFlt-1* mRNA could be detected at either stage (Figure 5D,G), indicating there is no endogenous inhibition of VEGFA signalling via sFlt-1 under these experimental conditions.

### VEGFA exerts neurogenic effects only in the absence of sFlt-1

**Figure 6:**
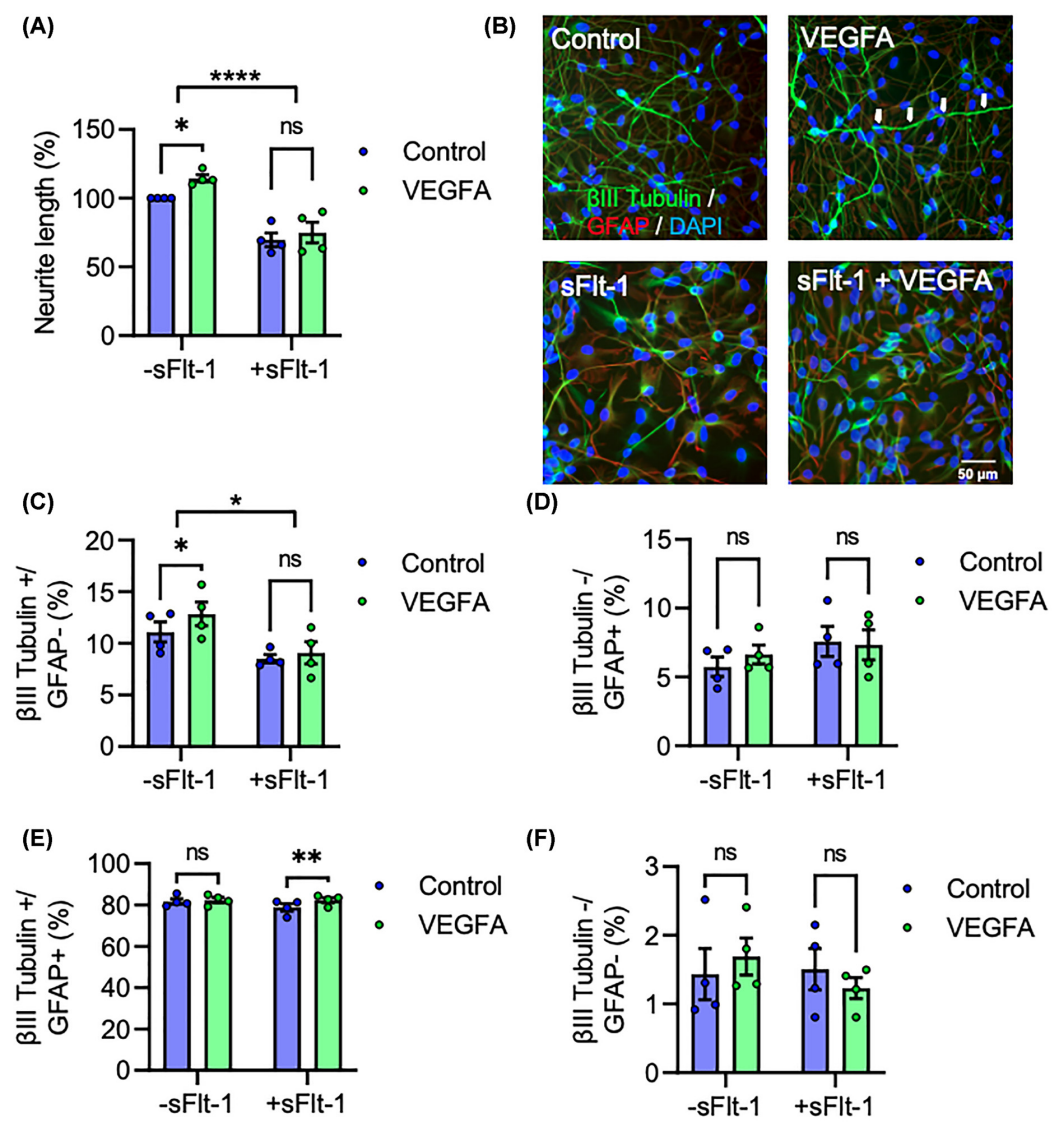
VEGFA exerts neurogenic effects only in the absence of sFlt-1 ReNcell® VM were differentiated into neurons and glia over 15 days while exposed to 100 ng/ml sFlt-1, 50 ng/ml VEGFA, both sFlt-1 and VEGFA, or PBS as vehicle control. (**A,B**) Graph and representative photomicrographs of neurite length. (**C–F**) Graphs of βIII Tubulin and GFAP protein expression. Data are mean + SEM from four independent experiments (*n*=4) expressed as percentage of control for (A) or as percentage positive cells for (C**–**F). Two-way ANOVA and post-hoc Fisher’s least significant difference (LSD) test (*P<0.05, **P<0.01, ****P<0.0001 vs. control).

To determine if sFlt-1 inhibits the neurogenic effects of VEGFA *in vitro*, ReNcell® VM were treated with VEGFA, with or without sFlt-1 co-treatment, for 15 days’ differentiation. VEGFA increased neurite growth, but not in the presence of sFlt-1. A two-way ANOVA revealed a significant main effect of sFlt-1 in reducing neurite growth (F_1,12_ = 56.58, *P*<0.0001), while post-hoc analysis revealed a significant increase with VEGFA treatment, only in the absence of sFlt-1 (*P*<0.05) (Figure 6A,B). In line with this increase in neurite length, VEGFA also promoted neuronal differentiation only in the absence of sFlt-1–sFlt-1 reduced the overall proportion of cells expressing βIII-tubulin but not GFAP after 15 days (F_1,3_ = 10.14, *P*<0.05), which was increased by VEGFA (*P*<0.05) only when sFlt-1 was not present (Figure 6B,C). Neither sFlt-1 nor VEGFA affected the number of βIII-tubulin-/GFAP+ (Figure 6D) or Tubulin-/GFAP- (Figure 6F) cells, although VEGFA did lead to a small increase the number of cells expressing both proteins (F_1,3_ = 12.58, *P*<0.05), specifically in the presence of sFlt-1 (*P*<0.05) (Figure 6E). Overall, these data are strongly in line with the neurite length and differentiation data presented above, and demonstrate that while VEGFA promotes neurite growth and neurogenesis, it is unable to do so in the presence of sFlt-1.

## Discussion

Exposure to PE increases the risk of neurodevelopmental disorders in exposed offspring, suggesting that PE may impact neurodevelopmental processes in the foetal brain, although the molecular mediators are largely unknown. As PE is characterised by elevated sFlt-1, here we tested the hypothesis that sFlt-1 may have a direct anti-neurogenic role on developing human neurons. In support of this proposal, we found that exposure to 100 ng/ml sFlt-1 during differentiation led to a ∼25–30% reduction in neurite length in both SH-SY5Y cells and ReNcell® VM-derived neurons. Interestingly, the effect was also timing-dependent, in that exposure to sFlt-1 was required from the beginning of the 15-day differentiation process to elicit an effect, thus demonstrating that sFlt-1 inhibits the growth, but not the maintenance of established neurites. This notion is supported by the finding that sFlt-1 reduces neurite length of neurospheres, but only if it is present in the culture medium from the beginning of the experiment, and further by the findings of Licht et al. (2010) who report that sFlt-1 overexpression in mice impairs dendrite growth and spine number in newly born, but not mature adult olfactory neurons, and is mechanistically plausible given that the initial growth and retraction of neurites can be governed by different processes [43]. Interestingly, in the case of neurospheres in the current study, early treatment with sFlt-1 reduced neurite length even after it was removed at the beginning of differentiation, implying that neurite growth mechanisms do not recover after this early exposure, although a longer experiment would be required to discern if this is indeed the case. These findings are in line with the putative mechanisms of VEGFA inhibition, given that VEGFA increases neurite length *in vitro* [35,36]. Although the concentration of 100 ng/ml is close to physiological amniotic fluid concentrations in PE [21], physiological cord blood concentrations, while highly elevated in PE compared with uncomplicated pregnancy, are reported as 0.25–5 ng/ml [21,31,44,45]. While an equivalent dose of 1 ng/ml did reduce neurite length in our study by ∼25%, this was not statistically significant (*P*=0.08), and all further experiments used a single concentration of 100 ng/ml.

sFlt-1 did not affect neurosphere size, which appears contradictory to reports that VEGFA increases NPC proliferation, while sFlt-1 decreases proliferation [32,46]. However, neurosphere size alone is not equivalent to proliferation, which is governed by additional factors such as the number and viability of spheres. In fact, after 3–4 DIV, sphere size becomes negatively correlated with the number of viable cells [47]. On the other hand, sFlt-1 significantly reduced the expression of transcripts for *βIII-tubulin*, without any significant effect on *nestin*, or glial markers *GFAP* or *MBP*. Human neurons and glia co-express βIII-tubulin and GFAP at intermediate and immature stages of differentiation [48,49], and they remain co-expressed by many cells in differentiated cultures [50,51]. Therefore, restricting neurite length and neurogenesis measurements to βIII-tubulin+/GFAP- cells in the current study ensured only mature neurons which have survived this intermediate stage and no longer expressed GFAP were quantified. sFlt-1 reduced the proportion of cells in differentiated neurospheres expressing βIII-tubulin but not GFAP, indicating sFlt-1 inhibits neuronal differentiation. As with the reduction in neurite length, this decrease in neuronal differentiation is highly concordant with a hypothesised mechanism of VEGFA inhibition, with previous reports demonstrating a pro-neurogenic effect of exogenous VEGFA [35,37].

**Figure 7:**
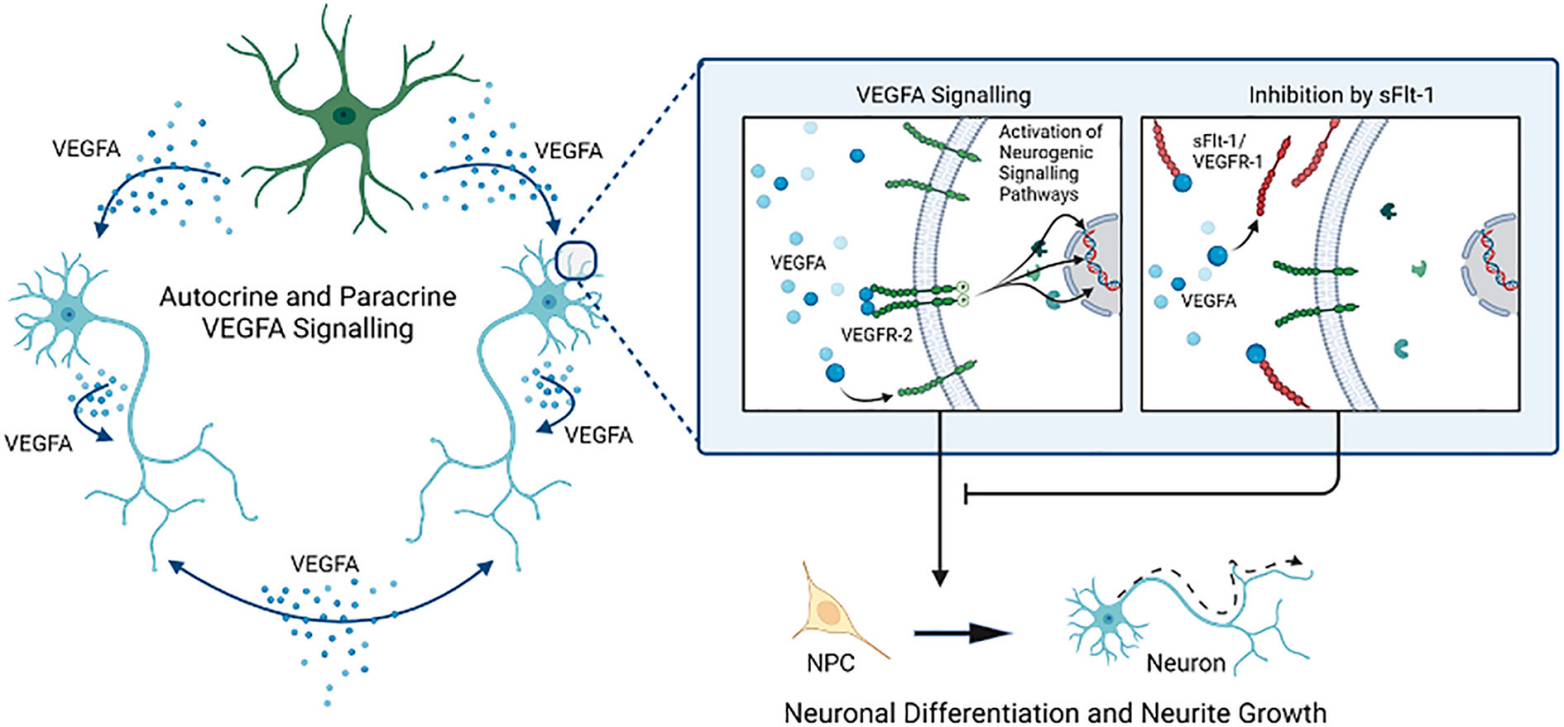
Summary of findings and proposed mechanism sFlt-1/VEGFR-1 reduces neurite growth and neuronal differentiation in human neural progenitor cells, whereas VEGFA increases neurite length and neuronal differentiation, but only in the absence of sFlt-1. ReNcell® VM express VEGFA and VEGFR-2, which modulate autocrine and paracrine VEGFA signalling, both as undifferentiated neural progenitors and as a mixed culture of neurons and glia. Crucially, there is no expression of sFlt-1 mRNA for endogenous inhibition of VEGFA signalling. Therefore, exogenous sFlt-1 is likely eliciting its anti-neurogenic effects by binding VEGFA and inhibiting endogenous VEGFA signalling.

Both undifferentiated NPCs and differentiated neurons and glia express high levels of *VEGFA* and *VEGFR-2* mRNA, the ligand and receptor pair required for autocrine and paracrine VEGFA signalling. Considering VEGFA is not a constituent of the culture media used in this study, any VEGFA found in the culture must be synthesised *de novo*, providing the first human neuron corroboration of the finding that rodent neurons engage in autocrine and paracrine VEGFA signalling independently of endothelial cells [35,37]. Importantly, *Flt-1/VEGFR-1* mRNA was not detectable at either DIV1 or DIV 15, and therefore the only sFlt-1 present was that added exogenously. This is consistent with previous evidence that while rat hippocampal NPCs and cortical neurons express VEGFA and VEGFR-2, neither cell type expresses VEGFR-1 [32,36]. Finally, while VEGFA is known to elicit a pro-neurogenic effect on developing neurons, we observed that this is absent in the presence of sFlt-1. Given these data, we postulate that exogenous sFlt-1 reduces neurite growth and neurogenesis in human neurons via inhibition of endogenous neurogenic autocrine and paracrine VEGFA signalling (Figure 7).

While the present findings have implications for our understanding of the relationship between PE and neuron development, it is important to consider them within their *in vivo* context, which is markedly more complex. The most conspicuous feature that distinguishes the *in vitro* from the *in vivo* context is the influence of the cerebrovasculature. The anti-angiogenic role of sFlt-1 is well-described, and if insufficient vascular development results in local hypoxia, this can up-regulate neuronal VEGFA expression [52]. Furthermore, not only does vascular-derived VEGFA promote neurogenesis, but the relationship is reciprocal, in that neuron-derived VEGFA promotes local blood vessel formation via angiogenesis [34,37]. Secondly, there is the varying involvement of different cell types. Licht (2010) and Okabe (2020) report that VEGFR-2 is not highly expressed by mouse olfactory, hippocampal, or cortical neurons, but is instead expressed by local endothelial and ependymal cells, raising the possibility of indirect paracrine signalling through these other cell types. VEGFA also regulates glial development, by promoting oligodendrocyte precursor cell migration and astrocyte proliferation [46,53]. Additionally, certain subpopulations of adult neurons express transmembrane VEGFR-1, which can transduce signals in certain situations, such as sensory neurons in the context of neuropathic pain [54,55]. Thirdly, elevated sFlt-1 in PE cannot be seen as independent from other aspects of the disorder, most prominently maternal immune activation. Astrocytes and monocytes both increase sFlt-1 expression in response to cytokine stimulation [56,57], while sFlt-1 in turn modulates cytokine expression [58,59] and chemotactic migration of peripheral monocytes and microglia [60,61]. Therefore, local and peripheral immune cells are likely to exacerbate the anti-angiogenic and anti-neurogenic roles of sFlt-1, adding to the complexity of the *in vivo* environment. Lastly, it is important to consider that PE is generally diagnosed after 20 weeks’ gestation, when neuronal differentiation (and, to a lesser degree, neurite growth), is mostly already established in humans. Interestingly, sFlt-1 is in fact elevated in the first trimester of mothers who are only later diagnosed with PE [62], when neuronal differentiation and neurite growth are highly dynamic, although the difference in concentration is notably less than that in late pregnancy, and it is unclear whether this smaller difference in the first trimester is sufficient to elicit the single-cell effects reported in the current study. These factors should be considered when interpreting the effects of sFlt-1 in neurons.

The current study presents the first direct evidence that exogenous sFlt-1 reduces neurite growth and neurogenesis in human neurons. A significant strength of this work is the use of human NPCs and their differentiation into mature human neurons and glia. Further strengths include the concordance of different observations – neurite length findings were replicated in SH-SY5Y and ReNcell® VM; reduction in *βIII-tubulin* mRNA was confirmed by immunostaining; and the putative mechanism of VEGFA signalling is supported by *VEGFA* and *VEGFR-2*, but not *VEGFR-1*, mRNA expression at DIV1 and DIV15, and by sFlt-1 blocking the neurogenic effects of exogenous VEGFA. Nonetheless, there are some important limitations to this work. While the current study aimed to characterise the effects of sFlt-1 at a single cell level, the interpretation of these findings must take into account the increasingly complicated situation *in vivo*. Additionally, downstream signalling pathways were not explored, although VEGFR-2 is known to dimerise and autophosphorylate upon ligand binding to transduce signals predominantly through PLCγ and PKC [30,63], from which point there are well-characterised mechanisms of neurite growth and neurogenesis, such as activation of MAPK/ERK and STAT3 signalling [37], and epigenetic regulation such as inactivation of histone deacetylase 5 [64]. Future work should aim to characterise whether manipulation of these pathways can attenuate the anti-neurogenic effects of sFlt-1. Lastly, the precise role of astrocytes could be explored further, by selective knockdown of *VEGFA* and *VEGFR-2* in neurons or astrocytes only. Overall, it is clear from the present findings that sFlt-1 impairs neuronal development independently of its effects on the vasculature, and in that regard may be considered an anti-neurogenic, as well as an anti-angiogenic, factor. Given that the foetal brain is likely to be exposed to elevated and sustained sFlt-1 concentrations during PE, these findings may provide important insights into the potential pathogenic mechanisms mediating the relationship between the disorder and the sub-optimal neurodevelopmental trajectories of exposed offspring.

## Data Availability

All data generated during this study are included in this article and all datasets from which conclusions are based are available on reasonable request from the corresponding authors.

## Abbreviations

ADHD: Attention-deficit/hyperactivity disorder
ASD: Autism spectrum disorder
BSA: Bovine serum albumin
DIV: Days *in vitro*
DMEM: Dulbecco’s modified Eagle’s mixture
EGF: Epidermal growth factor
FGF2: Fibroblast growth factor 2
GFAP: Glial fibrillary acidic protein
HDP: Hypertensive disorder of pregnancy
ID: Intellectual disability
LDH: Lactate dehydrogenase
LSD: Least significant difference
MBP: Myelin basic protein
NPC: Neural progenitor cell
PBS: Phosphate-buffered saline
PE: Pre-eclampsia
PFA: Paraformaldehyde
PlGF: Placental growth factor
RA: Retinoic acid
SEM: Standard error of the mean
sFlt-1: Soluble Fms-like tyrosine kinase 1
VEGFA: Vascular endothelial growth factor A
VEGFR: Vascular endothelial growth factor receptor
